# Cardiac magnetic resonance T1 mapping pre and post contrast in heart transplant patients with clinical antibody-mediated rejection: a preliminary experience

**DOI:** 10.1186/1532-429X-16-S1-P100

**Published:** 2014-01-16

**Authors:** Emmanuelle Vermes, Julien Pucheux, Anne Delhommais, Daniel Alison, Laurent Brunereau

**Affiliations:** 1 Cardiac Imaging, Chu Trousseau, Tours, France

## Background

Antibody-mediated rejection (AMR) is characterized by histopathological and immunophenotypic findings such as activated endothelial cells, intravascular macrophages and evidence of capillary C4d deposition. This inflammatory reaction could be followed by diffuse fibrosis. Cardiac magnetic resonance (CMR) with recently T1 mapping is a promising technique to identify diffuse myocardial fibrosis. The purpose of this study was to assess T1 mapping in patients with AMR.

## Methods

Two patients with clinical AMR (histopathological and immunophenotypic findings, presence of donor-specific allo antibodies and allograft dysfunction) performed a CMR study one week (for the first patient) and 3 weeks (for the second patient) after the treatment of AMR (plasmapheresis, IV Immunoglobulins and Rituximab). Images were acquired on a 1.5 Tesla scanner (Siemens) including T1 mapping using a shortened modified look-locker inversion-recovery sequence and T2 mapping in a matched mid-ventricular short axis slice using a black- blood single shot fast spin echo pulse sequence. Segmental and global T1 values were measured before and 15 minutes after administration of 0.2 mmol/kg of Gadoteric acid and compared to our cohort of 17 controls.

## Results

Mean non contrast T1 values were significantly higher in heart transplants patients compared to controls (1100 ± 5 ms vs 947 ± 29 ms, P < 0.001). Segmental T1 values were significantly higher in the 6 regions of interest compared to controls (P < 0.001 in all segments). Mean post contrast T1 values were not significantly different in patients and controls. Mean T2 value was higher in patients compared to controls (73 ± 13 vs 50 ± 4 ms), suggesting the presence of global edema.

## Conclusions

Heart transplant patients with clinical antibody-mediated rejection show a significant increased global and segmental non contrast T1 values suggesting the presence of diffuse myocardial fibrosis. Further studies are required to confirm these data.

## Funding

No funding.

